# Integrated carbon capture and conversion: A review on C_2+_ product mechanisms and mechanism-guided strategies

**DOI:** 10.3389/fchem.2023.1135829

**Published:** 2023-02-16

**Authors:** Asmita Jana, Seth W. Snyder, Ethan J. Crumlin, Jin Qian

**Affiliations:** ^1^ Chemical Science Division, Lawrence Berkeley National Laboratory, Berkeley, CA, United States; ^2^ Advanced Light Source, Lawrence Berkeley National Laboratory, Berkeley, CA, United States; ^3^ Energy & Environment S&T, Idaho National Laboratory, Idaho Falls, ID, United States

**Keywords:** one-pot solution, carbon capture, carbon conversion, C_2+_ products, electrochemical reduction, metal organic framework, copper catalysts

## Abstract

The need to reduce atmospheric CO_2_ concentrations necessitates CO_2_ capture technologies for conversion into stable products or long-term storage. A single pot solution that simultaneously captures and converts CO_2_ could minimize additional costs and energy demands associated with CO_2_ transport, compression, and transient storage. While a variety of reduction products exist, currently, only conversion to C_2+_ products including ethanol and ethylene are economically advantageous. Cu-based catalysts have the best-known performance for CO_2_ electroreduction to C_2+_ products. Metal Organic Frameworks (MOFs) are touted for their carbon capture capacity. Thus, integrated Cu-based MOFs could be an ideal candidate for the one-pot capture and conversion. In this paper, we review Cu-based MOFs and MOF derivatives that have been used to synthesize C_2+_ products with the objective of understanding the mechanisms that enable synergistic capture and conversion. Furthermore, we discuss strategies based on the mechanistic insights that can be used to further enhance production. Finally, we discuss some of the challenges hindering widespread use of Cu-based MOFs and MOF derivatives along with possible solutions to overcome the challenges.

## Graphical Abstract

**Figure:**
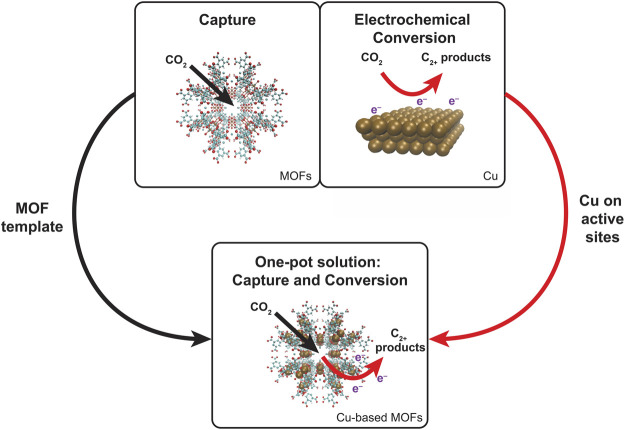
A one-pot solution of combined capture and conversion represents the ideal solution with the MOFs component aiding capture and the Cu constituents supporting conversion to C_2+_ products *via* CO_2_ER.

## 1 Introduction

The exponential increase in greenhouse gases (GHGs) including CO_2_ since the industrial revolution is the key driver of climate change (Field and Barros, 2014; Sun et al., 2017). Currently, the concentration of CO_2_ in the atmosphere is greater than 400 ppm (Jiao et al., 2019) with fossil fuels accounting for 75% of the increase in anthropogenic CO_2_ emissions (Field and Barros, 2014; Rafiee et al., 2018). CO_2_ levels are projected to increase to 685 ppm by 2050 (OECD, 2012) but CO_2_ levels need to be maintained at ≤ 450 ppm to avoid seriously jeopardizing the environment (Metz, 2007). While replacing fossil fuels with renewable sources of energy is the long-term solution, capturing CO_2_ from concentrated sources and from the atmosphere is urgently needed (Irena, 2018; Birol, 2019; Gielen et al., 2019). Three main separation technologies to capture CO_2_ are liquid absorbents, solid adsorbents, and membranes (‘Paul and Michelle, 2020: Report of the Basic Energy Sciences Workshop for Carbon Capture: Beyond 2020’, 2020) but the only method currently being used in an industrial scale is absorption using aqueous amine solutions (Iyer et al., 2017). However, it is fraught with issues like solvent losses and comes with a high sorbent regeneration cost. Thus, there is an ongoing search for better materials (and processes) that can achieve efficient and cost-effective carbon capture (‘Paul and Michelle, 2020: Report of the Basic Energy Sciences Workshop for Carbon Capture: Beyond 2020’, 2020). In this review, we evaluate a class of solid adsorbents in more detail- Metal Organic Frameworks (MOFs). These crystalline porous materials are composed of multi-metallic units surrounded by organic linkers (Eddaoudi et al., 2001; Long and Yaghi, 2009; Zhou et al., 2012; “Joe” Zhou and Kitagawa, 2014; Lu et al., 2014) and satisfy some of the prerequisites of an ideal CO_2_ capture material: a. high selectivity towards binding CO_2_, b. high capacity, c. low energy requirement for releasing CO_2_, d. thermal and chemical stability along with thermal capacity, and e. good synthesizability.

The step following carbon capture is either storage or conversion. Limited known geological storage options along with associated costs of transportation and suffocation risks from potential leaks suggest that conversion and utilization should be strongly committed (Sabri et al., 2021). Conversion achieves the dual objective of converting CO_2_ to value-added products including urea, methane, methanol, etc., while simultaneously avoiding the risks associated with storage. Conversion of CO_2_ invariably translates to CO_2_ reduction. Most thermal CO_2_ reduction requires harsh conditions involving high temperature and pressures, requires additional energy input and can hinder broad deployment. Depending on the energy source, it could require additional carbon-emitting fossil combustion. This can be circumvented by using CO_2_ electrochemical reduction (CO_2_ER) instead of thermal and pressure-driven methods (Kondratenko et al., 2013; Lu et al., 2014; Zhao et al., 2017; Jiao et al., 2019; Wang et al., 2022). Electrochemistry enables production of chemicals that are often more difficult to produce from thermal methods (Gattrell et al., 2006; Kondratenko et al., 2013). While a wide variety of value-added products can be obtained, not all of them are economically feasible. Nitopi et al. weighed the market price of an array of value-added products against the energy required to produce them and concluded that C_2+_ products such as ethanol, ethylene, and propanol are the most economically feasible (Nitopi et al., 2019). Hence, in this review, we shall focus on processes that exclusively produce C_2+_ products. Among the catalysts used for CO_2_ER, Cu-based ones are unparalleled for synthesis of C_2+_ products (Nitopi et al., 2019).

In this review, we focus on the conversion part of the capture and conversion process because the former is a process driven by thermodynamics and is a very well-studied topic on its own (Eddaoudi et al., 2001; Long and Yaghi, 2009; Zhou et al., 2012; Joe Zhou and Kitagawa, 2014; Lu et al., 2014). Here, we wanted to showcase the impact of electrocatalysis on CO_2_ reduction within the framework of a conversion material. We explore mechanisms for CO_2_ER to C_2+_ products in Cu-based electrodes to develop insights into the process. Such mechanistic knowledge shall guide different strategies that enhance C_2+_ product formation by optimizing the catalyst and reaction conditions. We then highlight the emerging potential of having a one-pot solution that combines capture and conversion. This has been demonstrated with Cu-based MOF derivatives where the constituent MOFs adsorb CO_2_ and provide a nice framework for constituent Cu sites to act as catalytic sites for CO_2_ER. Furthermore, the structure of MOF enhances catalytic activity *via* synergistic effects in addition to CO_2_ capture. While some of the mechanistic insights and improvement strategies in Cu electrocatalysis are transferable, we also discuss the additional requirements that are mandated by the one-pot Cu-based MOF solution. Finally, we discuss challenges hindering commercial use of these materials and how they can be overcome, paving the way for using these materials as suitable candidates for broad deployment of CO_2_ reduction systems.

## 2 CO_2_ conversion

Catalysts used to fabricate electrodes are broadly classified into four groups based on the selectivity of their CO_2_ reduction products: a. formate; b. CO; c. H_2_; and d. hydrocarbon, aldehydes, and alcohols. The formate producers are metals such as Pb, Hg, Tl, and In, while some metals such as Au, Ag, and Zn primarily produce CO. A few metals including Ni, Fe, Pt, and Ti reduce water to H_2_ and are termed H_2_ producing. Cu is the only metal belonging to the fourth category making it ideal for producing C_2+_ products. This is heavily attributed to the unique characteristic of Cu of having a negative adsorption energy for CO while maintaining a positive one for H species. This translates to the ability to retain and eventually reduce CO while preventing the hydrogen evolution reaction (HER). The adsorption characteristics of CO play an instrumental role because reduction to CO is the first reaction step in CO_2_ER to C_2+_ products (Nitopi et al., 2019). CO production is enhanced in the presence of catalysts such as Ag and they have been increasingly used to design bi-metallic catalysts with increased reduction to C_2+_ products (Nitopi et al., 2019).

**FIGURE 1:**
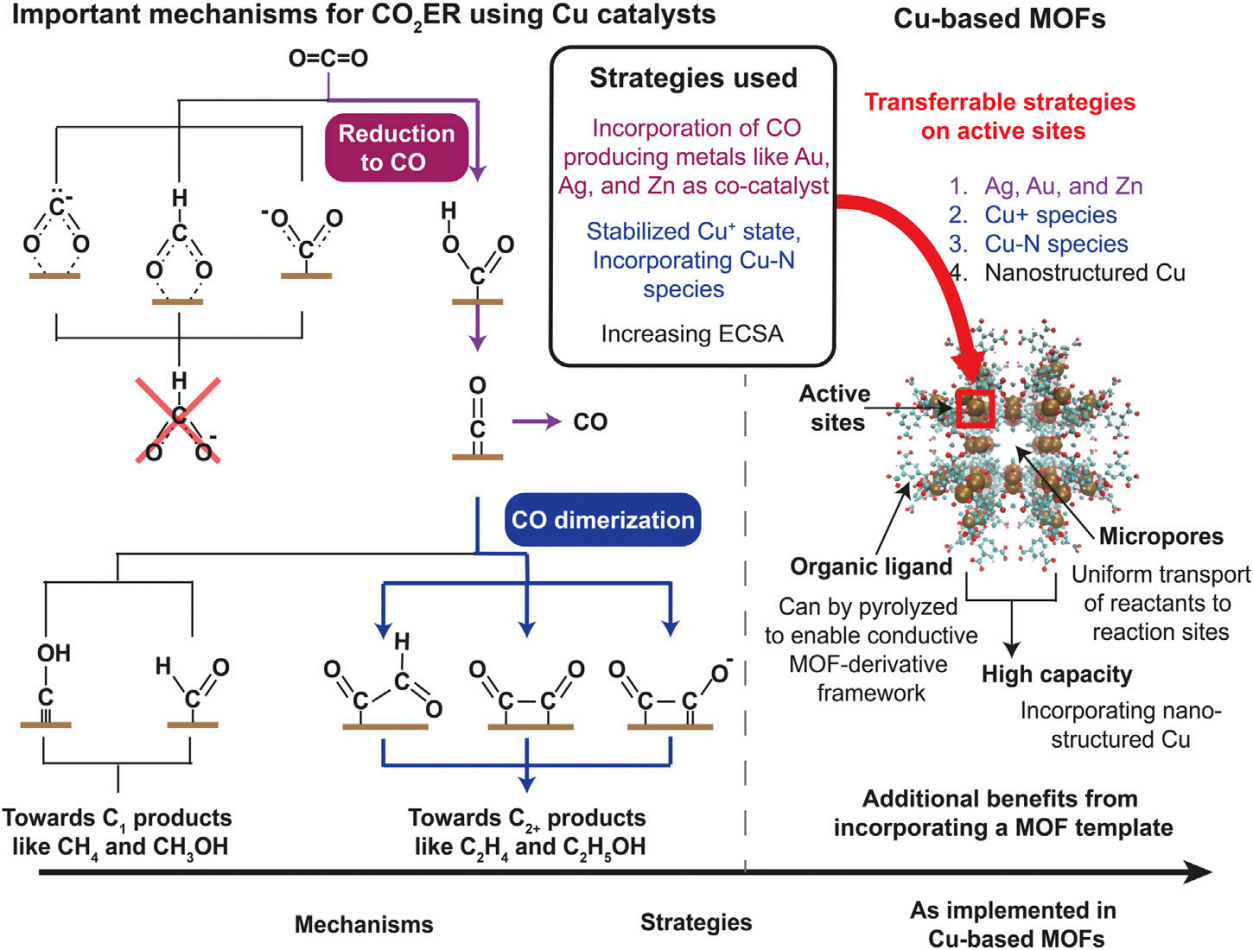
A partial CO_2_ER pathway indicating major reactions process (Hori et al., 1994; 1997; Peterson et al., 2010; Kuhl et al., 2012; Cheng et al., 2015; Kortlever et al., 2015; Montoya et al., 2015; Feaster et al., 2017; Chernyshova et al., 2018; Garza et al., 2018; Lum et al., 2018; Liu et al., 2019; Nitopi et al., 2019). Mechanisms that play a critical role in the production on C_2+_ products *via* CO_2_ER on Cu-based catalysts along with strategies used to enhance them are specifically highlighted. These strategies can be implemented in a Cu-based MOF system; the chemically tunable active sites along with additional advantages of a MOF template for catalysis highlights the ability of Cu-based MOFs to produce C_2+_ products *via* CO_2_ER.

While electrodes fabricated from Cu can synthesize a variety of products such as CO, formate, C_2_H_4_, and CH_4_, we are primarily interested in producing C_2+_ products given their high benefit-to-cost ratio. In specific, formation of the C-C bond in C_2+_ is the critical first reaction for oligomerization into a range of materials, chemicals, and fuels, enabling CO_2_ to serve as a primary industrial building block. Many processes exist to maximize the production of C_2+_ products but from a reduction pathway perspective, they broadly result in these effects: a. increasing the availability of CO and b. enhancing CO dimerization reactions. The first tier of products synthesized after two electron transfer from CO_2_ in the CO_2_ER reduction pathway consists of formate and CO (Nitopi et al., 2019). The former is a terminal product, implying that it does not undergo further reduction. Thus, for C_2+_ production, we need to drive the reduction process to CO, not formate. Increasing the adsorption, transport, and overall availability of CO can enhance formation of C_2+_ products. This is the principle behind employing metals that selectively produce CO such as Ag, Au, and Zn as a co-catalyst (Nitopi et al., 2019). For example, a study found Cu-Ag bimetallic electrodes resulted in higher production of CH_3_CHO and C_2_H_4_ compared to pure Cu electrode (Ishimaru et al., 2000). The second process is based on a reaction pathway that involves enhancing CO dimerization reactions (Nitopi et al., 2019). Cu (100) is observed to amplify those reactions and is reported to be one of the surfaces with a high selectivity for C_2_H_4_ over CH_4_ (Hori et al., 1995). There are other strategies to boost C_2+_ products, the most important and widespread one is increasing the electrochemically-active surface area (ECSA). Some methods for increasing ECSA include using Cu nanostructures and stepped Cu surfaces. For example, Manthiram et al. observed fourfold higher current density along with a faradaic efficiency of 80% for CH_4_ production on Cu nanoparticles with an initial size of 7 nm as compared to polycrystalline Cu foil (Manthiram et al., 2014). These processes are depicted in Figure 1.

## 3 Integrated CO_2_ capture and conversion

Carbon conversion is preceded by carbon capture, but such sequential treatment suffers from the additional cost required for compression and transportation. For transportation, CO_2_ needs to be compressed up to 150 bar which translates to at least around 360 kJ/kg CO_2_ of work (Huck et al., 2014). Other challenges with compression include the removal of residual moisture to avoid creating corrosive conditions in the tank and dissipation of heat during the compression process. These costs can be avoided if both processes occur in the same plant location, termed an integrated cascade system (Sabri et al., 2021). The extremely optimistic end of this spectrum is simultaneous capture and conversion. Often, this is achieved by materials that function as both CO_2_ adsorbers and CO_2_ reduction catalysts.

Interestingly, metal organic frameworks (MOFs) which belong to the category of solid adsorbers can serve as both an adsorbent for CO_2_ capture as well as a catalyst for CO_2_ conversion. Some of the attractive features of MOFs that make it ideal for CO_2_ capture include a. porous structure, b. chemically tunability, c. compatibility with other materials, d. structural flexibility, and e. hydrophobicity (Ding et al., 2019). The open metal sites and organic linkers serve as Lewis acid and basic sites, respectively, both of which can coordinate and bind CO_2_ (Ding et al., 2019). For example, M-MOF-74 (M: Mg, Ni, Co., Zn) coordinates CO_2_ *via* the metal sites (Kong et al., 2012; Queen et al., 2014) while IRMOF uses its N-containing Lewis base (amines) as the site for binding CO_2_ (Millward and Yaghi, 2005). Moreover, CO_2_ capture is enhanced with linkers functionalized with polar functional groups like -F, -Br, -NO_2_, and -SO_3_ as the additional dipole interacts with the quadrupole of CO_2_ (Debatin et al., 2010; Gassensmith et al., 2011; Zhang et al., 2016; Mosca et al., 2018; Ding et al., 2019). Additionally, the chemical features can be altered by easily functionalizing the linkers and modifying metal centers. These sites and modifications enhance CO_2_ capture *via* synergistic effects. Lastly, the pore size and flexibility of these hydrophobic structures are tunable (Ding et al., 2019).

Synthesizing C_2+_ products *via* electroreduction of CO_2_ calls for Cu-based electrodes to serve as catalysts. The easily tunable characteristic of MOFs translates to the ability to incorporate Cu within the framework (as shown in Figure 1). Cu-based MOFs such as HKUST-1 exist that exhibit selectivity for C_2+_ products when used as an electrochemical catalyst (Han et al., 2021a). Table 1 highlights the catalysts, products, and synthesis efficiency. More importantly, we believe that it is important to highlight that not only do Cu-based MOFs combine both capture and conversion capabilities, but the combination increases the performance of both processes.

**TABLE 1:** List of Cu-based materials used as electrodes for CO_2_ER to C_2+_ products.

Adsorber	Material category	Conductive component	Electrolyte	Reactor cell type	Potential (V vs. SHE)	Potential (V vs. RHE)	Products	Efficiency (%)	Current density (mA/cm^2^)	Reference
Cu	Metal	Metal	0.1 M KHCO_3_	H-type Pyrex cell	−1.44	−1.05	C_2_H_4_	25.5	−5	Hori et al., 1995; Hori (2008)
							C_2_H_5_OH	5.7		
							C_3_H_7_OH	3		
Cu (100)					−1.4	−1	C_2_H_4_	40.4		
							Alcohol	12		
							Aldehyde	4.4		
Cu (110)					−1.55	−1.15	C_2_H_4_	15.1		
							Alcohol	7.4		
							Aldehyde	3.1		
Cu (111)					−1.55	−1.15	C_2_H_4_	8.3		
							Alcohol	3.3		
							Aldehyde	2.7		
Cu-BTC	MOF	Glassy carbon electrode	0.1 M KCl	Three electrode singles compartment electrochemical cell	−2.27		Oxalic acid	51	19.22	Senthil Kumar, Senthil Kumar and Anbu Kulandainathan (2012)
Cu-BTC			0.5 M KHCO_3_	Micro Flow cell, ElectroCell A/S	−0.28		C_2_H_5_OH	10.3	10	Albo et al. (2017)
HKUST-1		Carbon paper	0.1 M KHCO_3_	H-type cell		−0.98	C_2_H_4_, C_2_H_5_OH	58.6	19.2	Han et al. (2021a)
MOF-Cu_3_(HITP)_2_		Conducting support	0.1 M KHCO_3_	Two compartment H-cell	−1.56		C_2_H_4_	48	48	Sun et al. (2021)
HKUST-1				Micro Flow cell, ElectroCell A/S			C_2_H_5_OH	15.9	10	Albo et al. (2017)
CuAdeAce								1.2		
CuDTA MOA								6		
CuZnDTA MOA								9.9		
CS^+^ modified Cu, Zn MOF (Zr_12_ BPDC)							C_2_H_5_OH			An et al. (2019)
Cu(II)/ade-MOF				H-type cell		−1.4	C_2_H_4_	34	8.5	Yang et al. (2019)
MOF-Cu_3_(HITP)_2_		Conducting support		H-cell, flow-cell			C_2_H_4_	60–70		Sun et al. (2021)
OD Cu/C HKUST-1	MOF derivatives *via* pyrolysis	Carbonized structure	0.1 M KHCO_3_	Two bath cell		−0.1	C_2_H_5_OH	45.2–71.2		Zhao et al. (2017)
HKUST-1 with desymmetrized Cu dimer			1 M KOH	Flow cell	−1.07		C_2_H_4_	45	262	Nam et al. (2018)
Cu MOF-NC; BEN-Cu BTC			0.1 M KHCO_3_	H-cell		−1.01	C_2_H_4_	11.2	8	Yao et al. (2020)
							C_2_H_5_OH	18.4		
Cu@CuxO-MOF HKUST-1			0.1 M KHCO_3_	Flow cell		−1.58	C_2_H_4_	51	150	Yao et al. (2020)
HKUST-MOF derived CuxOyCz			1 M KOH	Three compartment cell			C_2_H_4_, C_2_H_6_, C_2_H_5_OH, C_3_H_7_OH	54	80	Sikdar et al. (2021)
CuZn-NC MOF-74			0.1 M KHCO_3_	H-type compression cell		−1	C_2_H_4_, C_2_H_5_OH	25	12	Juntrapirom et al. (2021)
Cu_2_O/Cu@NC Cu-NBDC			0.1 M KHCO_3_			−1.6	C_2_H_4_	23.9		Jin et al. (2021)
Cu-GNC-VL			0.5 M KHCO_3_	H-type cell		−0.87	C_2_H_5_OH	70.53	10.4	Zhang et al. (2020)
Ag/Cu on HKUST			1 M KOH	Three compartment glass cell		−0.28	C_2_H_4_, C_2_H_6_, C_2_H_5_OH, C_3_H_7_OH	21	120	Sikdar et al. (2022)
Cu/Cu_2_O@NG (HKUST-1 and N-doped graphene)	MOF derivatives *via* electrochemical reaction	N-doped graphene	0.2 M KI	Single compartment gas diffusion cell		−1.9	C_2_H_4_, C_2_H_5_OH, C_3_H_7_OH	56	19	Zhi et al. (2021)
HKUST-1-derived Cu nanosheet		Cu foil	0.1 M KHCO_3_	H-type cell		−1.03	C_2_ products	56		Wang et al. (2021)

First, the availability of uniformly dispersed active sites and high surface area already makes MOFs an excellent catalyst template in general while the constituent micropores ensure uniform transport of reactants to the catalytic sites (Ding et al., 2019). MOFs have been used for thermal reduction of CO_2_ to synthesize organic products *via* cycloaddition, carboxylation, and cyclization reactions (Zalomaeva et al., 2013; Liu et al., 2015; Zhang et al., 2016; Liang et al., 2017; Xiong et al., 2017; Nguyen et al., 2018; Wang et al., 2018). For example, MMCF-2 has been shown to provide a 95% yield for cycloaddition of CO_2_ with propylene oxide in the presence of a co-catalyst (Gao et al., 2014).

Second, nanostructured Cu has been shown to display a higher catalytic activity compared to its bulk counterpart owing to a higher ECSA (Nitopi et al., 2019) arising from the structural increase in steps, facets, and edge atoms. Moreover, grain boundaries are found to promote C-C coupling reactions while suppressing HER (Sun et al., 2021). This effect can be readily exploited in MOFs and they provide an opportunity of significantly enhancing ECSA by incorporating Cu nanostructures and single atoms. For example, Cu-based MOFs such as HKUST-1 have been shown to produce C_2_H_4_ and C_2_H_5_OH at 59% efficiency (Han et al., 2021a). Moreover, a Cu-based HKUST-1 embedded on carbon catalysts *via* an electrochemical reaction resulted in ∼10-fold increase in faradaic efficiency for synthesizing C_2+_ products compared to a pristine Cu foil (Wang et al., 2021). This enhancement was attributed to the stepped surfaces, facets, and edges that contributed to the 1.24-fold higher ECSA observed compared to the Cu foil. Cu nanocrystallites were generated in MOF-Cu_3_(HITP)_2_ and the rich grain boundaries aided in C-C coupling reactions resulting in producing C_2_H_4_ with 60%–70% efficiency (Sun et al., 2021).

Third and most importantly, MOFs provide a structure where additional chemical species can be easily incorporated among the plethora of active sites available. From studies done on Cu electrodes, a variety of additions were found to enhance CO_2_ conversion that holds true for Cu-based MOFs as well, and are well-suited to be adapted within the MOF structure (Figure 1). One of the primary chemical modifications that enhances C_2+_ product formation is the addition of metals that produce CO such as Au, Ag, and Zn as co-catalysts. As mentioned in section 1, Au nanoparticles dispersed on a polycrystalline Cu foil results in a 100 x higher reduction rate to C_2+_ products compared to Cu alone (Morales-Guio et al., 2018). This effect is also observed in CuZn bimetallic material embedded in a carbonized MOF, where the influence of Zn led to a 5-fold increase in faradaic efficiency in producing C_2_H_4_ and C_2_H_5_OH (Juntrapirom et al., 2021). A similar effect was also seen in Ag/Cu bimetallic catalysts based on a Cu-based MOF derivative where many C_2+_ products were produced with an efficiency of 21% (Sikdar et al., 2022).

MOFs can also incorporate non-metals within its framework. Appending N-containing Cu-N species is credited to amplify formation of C_2+_ products and lend higher stability. The former is attributed to the more stable adsorption of the CH_2_ intermediate species and inhibition of HER (Jin et al., 2021). This is cited as the reason for the Cu_2_O/Cu@NC catalyst made from Cu-NBDC MOF showing 24% efficiency in forming C_2_H_4_ in direct contrast to the Cu_2_O/Cu@C catalyst (no N content) which did not produce any C_2_H_4_ (Jin et al., 2021). N, N dimethylformamide (DMF) was used in Cu-BTC and it was found to increase dimerization reactions producing oxalic acid with an efficiency of 51% (Senthil Kumar, Senthil Kumar and Anbu Kulandainathan, 2012). Similarly, N-containing ligands in Cu (II)/ade-MOFs augmented C_2_H_4_ generation with a 45% efficiency (Yang et al., 2019). An N-containing BEN-Cu-BTC derivative displayed efficiencies of 18% and 11% for C_2_H_5_OH and C_2_H_4_, respectively (Cheng et al., 2019). As seen from section 2, adding N-containing species is also a strategy used to increase CO_2_ capture as these groups interact with CO_2_ to enhance adsorption making this a dual-purpose addition for both capture and conversion.

Furthermore, stabilizing the Cu^+^ state was shown to boost synthesis of C_2+_ products *via* enhancing CO dimerization. This effect was observed in HKUST-1 where the high Cu^+^/Cu^0^ ratio resulted in producing C_2_H_4_ and C_2_H_5_OH with efficiency of 59% (Han et al., 2021a). For similar reasons, the Cu@Cu_x_O structure in a Cu-based MOF derivative created C_2_H_4_ with 51% efficiency (Yao et al., 2020).

While MOFs contain a plethora of advantages for CO_2_ER, they are also plagued by some disadvantages. Some of them are the lack of conductivity and stability. This necessitates the addition of conductive supports that can in turn, complicate manufacturability. To truly enhance the catalytic activity to the maximum limit, we need to explore methods of making the MOF conductive on its own. Fortuitously, this can be achieved by pyrolyzing the MOFs to create MOF derivatives that not only are stable and conducting because of the carbonization of the organic linkers at high temperatures, but the process also creates additional active sites (Chen et al., 2018). These reasons led to oxide-derived Cu/carbon catalyst generated from HKUST-1 *via* pyrolysis displaying a very low overpotential (−0.1 V vs. RHE) for production of C_2_H_5_OH (Zhao et al., 2017). Other such Cu-based MOF derivatives are listed in Table 1 with the products and their efficiencies. These examples demonstrate that Cu-based MOF derivatives are well suited for CO_2_ER to C_2+_ products.

## 4 Conclusion

**FIGURE 2:**
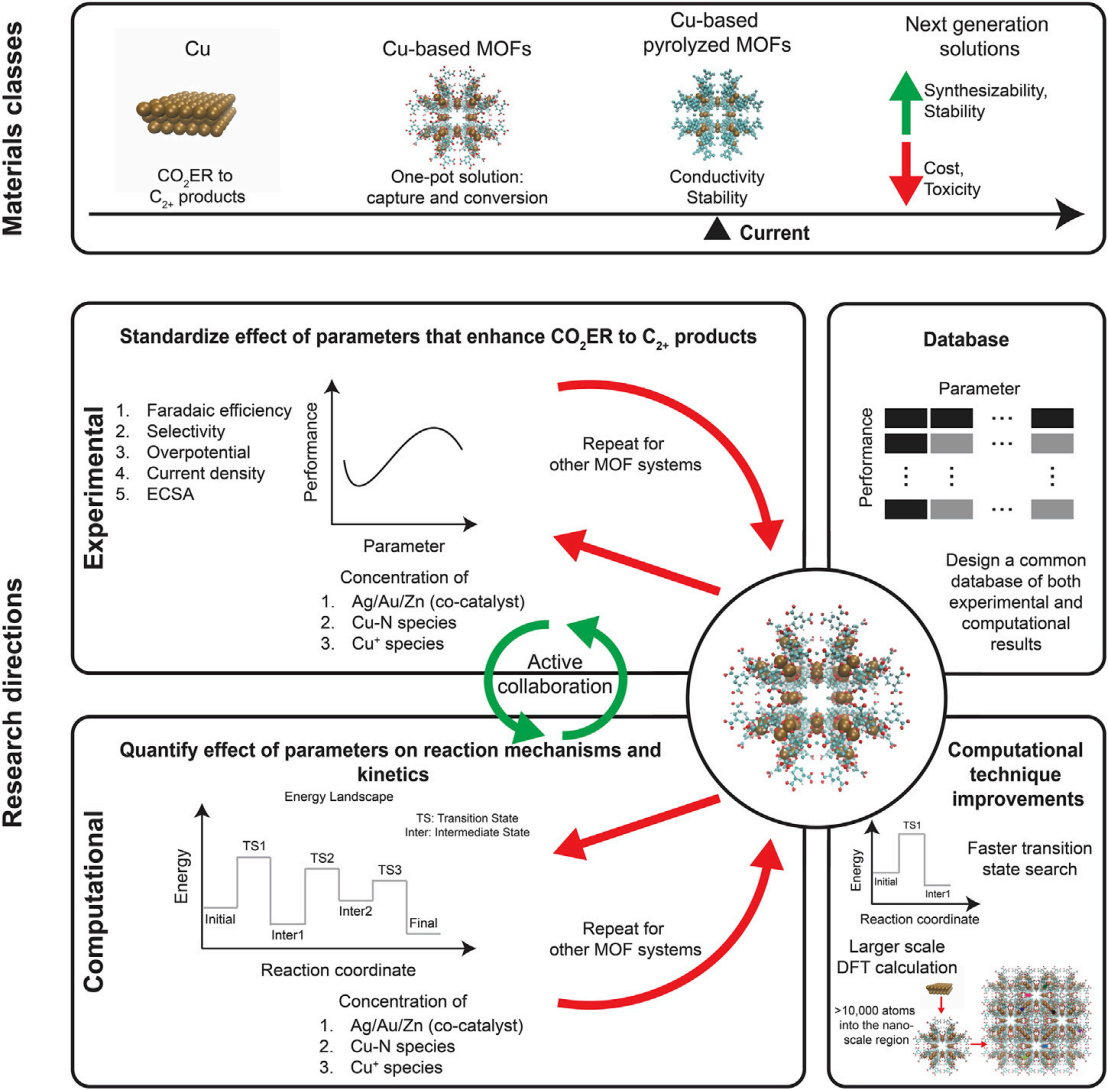
The materials classes involved in the CO_2_ER to C_2+_ products: each successive progression highlights an improvement over the prior group. Future research directions are depicted.

An integrated carbon capture and conversion process is urgently needed to address atmospheric GHG concentrations. A one-pot solution avoids the additional cost, energy burden, and risks of compression and transportation that is associated with the CO_2_ storage (and succeeding conversion). Only conversion to C_2+_ products is reported to be economically feasible. Since electrochemical reduction can be achieved under milder temperature and pressure conditions, they are preferred over thermal reduction. Cu-based catalysts are the gold standard for CO_2_ER to synthesize C_2+_ products while MOFs are renowned for their CO_2_ capture abilities. The chemical tunability of MOFs makes it possible to make Cu-based MOFs which fit perfectly with the requirement of the one-pot solution to enable CO_2_ capture and CO_2_ER synthesis of C_2+_ products. A series of Cu-based MOFs were explored and their performance in converting CO_2_ to C_2+_ products were summarized (Table 1). We also delved deeper into the mechanisms that are involved in C_2+_ product formation and various strategies that can be used to enhance it. Notable among these processes are incorporating Ag/Au/Zn as co-catalysts to increase CO yield, as converting CO_2_ to CO is the first step in the CO_2_ER pathway. Another strategy is increasing the ECSA of the catalyst, which is the principle behind using nanostructured Cu instead of polycrystalline Cu foil. Moreover, these Cu nanoclusters can be embedded into MOFs as well. We also explored one critical disadvantage of MOFs for electrocatalysis: their low electrical conductivity, which necessitates using conductive supports. However, using pyrolyzed MOFs is an easy way to achieve the requisite electrical conductivity while also boosting stability. These materials classes are highlighted in Figure 2. We believe that this one-pot solution can be used for cleaned and reacted flue gas with the added advantage of avoiding energies involved in pressurization and collection. This has been reported in a few studies (D’Alessandro et al., 2010; Kong et al., 2012; McDonald et al., 2012). Moreover, we want to highlight that this technology is in its conceptual stage and is not a working technology yet. Transformation to a full-fledged technology would require detailed evaluation of the scientific and engineering challenges such as separation of the reduction product from electrolyte and optimizing extended operation. A technoeconomic analysis would be useful to determine the scalability and to direct the implementation towards viable pathways.

While Cu-based MOFs display a high potential for CO_2_ER, translating it to commercial use requires us to overcome challenges associated with the scaling-up of fabrication and deployment of MOFs. Firstly, MOFs are expensive and often, the primary contributing factor is the solvent cost (DeSantis et al., 2017). For example, it accounts for 40% of the cost in HKUST-1 made *via* solvothermal process (DeSantis et al., 2017). Opting for alternative processes such as liquid assisted grinding (LAG) and aqueous synthesis has the potential to reduce the cost by 34%–83% as these processes use less solvent in comparison (DeSantis et al., 2017). Moreover, the presence of inorganic nano-sized metal ions that are non-biodegradable along with trapped non-aqueous solvents such as dimethylformamide (DMF) make MOFs toxic (Kumar et al., 2019). More detailed studies on the toxic effects of MOFs are required for design of less toxic MOFs. Additionally, operation of MOFs for electrocatalytic applications often subject them to exposure to harsh chemical environments making chemical stability essential for efficient operation. Stability, in turn, is a direct result of the bonding environment including interactions such as hydrogen bonding and pi stacking (Coe, 2002; Nouar et al., 2008; Howarth et al., 2016). Incorporating a more inert central metal ion in the MOF structure can increase chemical stability (Kang et al., 2011; Leus et al., 2016). MOF derivatives synthesized *via* pyrolysis also slow down metal nanoparticle corrosion in the electrolyte, which in turn improves stability (Xiao et al., 2022). Another strategy to consider would be wrapping a protecting film on the surface of MOFs and MOF derivatives (Xiao et al., 2022). Mechanical stability is another factor affecting operation and while strategies like interweaving MOF networks (Tan and Cheetham, 2011; Burtch et al., 2014; Katz et al., 2015) are often used, this can be redundant in MOFs for CO_2_ER as liquid electrolyte filling MOFs enhances its stability already (Bennett et al., 2015; van de Voorde et al., 2015). Thermal stability is not as important in MOFs for CO_2_ER as electroreduction does not require harsh thermal conditions for CO_2_ capture or conversion. Finally, the poor electrical conductivity of the organic linkers necessitates presence of conductive substrates such as carbon cloth (Xiao et al., 2022). As previously highlighted, MOF derivatives created *via* pyrolysis can provide the required conductivity with the formed carbon layers. By altering synthesis parameters, the degree of graphitization can be enhanced. By adjusting the chemistry of the MOF precursors, the degree of defect carbon structure can be tuned. Both strategies can be used to optimize the resulting MOF derivative for enhanced conductivity (Xiao et al., 2022).

Lastly, we summarize our review with Figure 2 and we provide conjectures regarding research needs for the next-generation one-pot carbon-neutral solutions. Future research should standardize experimental results to facilitate comparison across multiple studies, enabling the community to accelerate the discovery process (Ward et al., 2022). Most of the electrochemical reductions occur at the interface of the electrode and the electrolyte. Using experimental characterization techniques can help us understand bonding and identify species participating in the reaction. Overall, investigating the solid/liquid interface using characterization techniques requires overcoming two major challenges. First, characterization techniques need to be adapted for high pressure environments, as opposed to high vacuum, to replicate the more realistic experimental conditions. Second, the region corresponding to the electrical double layer is small and sandwiched between the bulk electrode and the bulk electrolyte, requiring for Å to nm resolution. Thus, the characterization techniques need to be specifically modified to enable a focus on the solid/liquid interface. Recent developments in characterization techniques like Fourier Transform Infrared spectroscopy (FTIR), Raman spectroscopy, and Ambient-Pressure X-Ray Spectroscopy (APXPS) have been able to overcome these challenges (Tian & Ren, 2004; Zaera, 2014; Favaro et al., 2017b; Salmeron, 2018; Lu et al., 2019; Han et al., 2021b; Radjenovic et al., 2021; Ye & Liu, 2021; Hao et al., 2022). Using these characterization tools could enable a deeper understanding of the mechanisms involved in the activation process (Favaro et al., 2017a; Qian et al., 2019; Qian et al., 2020). The field will benefit from active collaboration across experimental and computational investigations with the former focused on evaluating the performance of Cu-based MOFs as a function of parameters involved in the mechanism enhancement. On the other hand, computational studies can play a key role in assessing the energy landscape involved in the conversion of CO_2_ to specific products. These assessments can be accelerated by implementing larger scale density functional theory (DFT) calculations (Kronik et al., 2006; Zhou et al., 2006; Xu et al., 2019; 2021; Motamarri et al., 2020; Dogan et al., 2022; Hao et al., 2022; Xu et al., 2022) along with a faster transition state search procedure (Koistinen et al., 2017; Garrido Torres et al., 2019; Meyer et al., 2019). Eventually, a database common to both experimental and computational work, as outlined by the Materials Project (Jain et al., 2013) can be constructed which can go a long way towards helping design high performance solutions.

## Abbreviations

DOBDC; 2,5-Dihydroxyterephthalate, BTC; Benzene-1,3,5-tricarboxylate, BDC; 1,4-Benzenedicarboxylate, BPDC; Biphenyl-1,4-dicarboxylate, M-MOF-74; [M_2_(DOBDC) (H_2_O)_2_] (M = Mg^2+^, Ni^2+^, Co^2+^, Zn^2+^), IRMOF-3; [Zn_4_O(NH_2_BDC)_3_], HKUST-1; [Cu_3_(BTC)_2_(H_2_O)_3_], HITP; 2,3,6,7,10,11-hexaiminotriphenylene, H-CuTCPP; Helical Cu porphyrinic MOF meso-tetra (4- carboxyphenyl) porphyrin, CuAdeAce; Copper(II)–adeninate–acetate, CuDTA MOA; Copper bis-bidentate dithiooxamidate metal-organic aerogel, Cu(II)/ade-MOF; CuII/adeninato/carboxylato metal-biomolecule frameworks, Cu MOF-NC; BEN-Cu BTC; Calcination of N-containing benzimidazole-modified Cu-BTC MOFs (BEN-Cu-BTC), Cu-NBDC; Cu 2-aminoterephthalic acid, Cu-GNC-VL; Cu/Cu_2_O nanocomposite loaded on the surface of carbon derived from direct carbonization of two-dimensional cross-like zeolitic imidazolate framework-L coated vertically on graphene oxide, H_4_TACTMB; 1,4,7,10-tetrazazcyclododecane-N,N′,N″,N‴-tetra-p-methylbenzoic acid, MMCF-2; [Cu_2_(Cu-TACTMB) (H_2_O)_3_(NO_3_)_2_], F-IRMOF-3; Functionalized IRMOF; NH_2_ groups converted to quaternary ammonium salt though introduction of CH_3_I.
